# Beyond Diagnostic Overshadowing: Understanding Schizophrenia in the Population with Intellectual Disability

**DOI:** 10.1192/j.eurpsy.2026.11250

**Published:** 2026-07-31

**Authors:** P. M. Martins, A. Samouco

**Affiliations:** Department of Psychiatry, ULS Tâmega e Sousa, Porto, Portugal

## Abstract

**Introduction:**

Intellectual disability (ID) affects approximately one percent of the population. The relationship between ID and schizophrenia is complex. Individuals who later develop schizophrenia tend to exhibit lower intelligence quotient scores, with a further decline often occurring during the prodromal phase preceding illness onset. Moreover, offspring of individuals with schizophrenia have an increased risk of developing ID.

**Objectives:**

To synthesize current evidence on the prevalence, risk factors, diagnostic challenges, and prognostic outcomes of schizophrenia among individuals with ID.

**Methods:**

Narrative literature review.

**Results:**

Schizophrenia is three times more prevalent in adults with ID than in the general population. This elevated prevalence may be attributed to shared genetic vulnerabilities and environmental factors, particularly obstetric complications. Accurate diagnosis of schizophrenia in individuals with ID poses significant challenges, as it requires a level of communicative ability that many lack. In those with severe or profound ID, the assessment of reality testing is often unreliable, rendering diagnosis particularly difficult. Core features such as disorganized thinking may be difficult to identify, and behaviours such as talking to oneself, interaction with inanimate objects, or abnormal postures can overlap with the behavioural phenotype of ID. Additionally, individuals with ID may experience visual rather than auditory hallucinations, potentially reflecting underlying neurobiological differences. Adults with comorbid ID and schizophrenia tend to exhibit more pronounced negative symptoms, higher rates of treatment resistance, and greater functional impairment compared to those with schizophrenia alone.

**Conclusions:**

The increased prevalence of schizophrenia among individuals with ID carries significant implications for clinical practice. Diagnostic overshadowing, where behavioural disturbances are attributed solely to ID, leading to missed comorbid diagnoses, remains a major challenge in this population. As schizophrenia is a potentially treatable disorder, timely recognition and effective management are essential to alleviate distress and improve quality of life in individuals already burdened by substantial disability.

**Disclosure of Interest:**

None Declared

